# Distinct expression profiles and functions of Kindlins in breast cancer

**DOI:** 10.1186/s13046-018-0955-4

**Published:** 2018-11-26

**Authors:** Paula Azorin, Florian Bonin, Ahmad Moukachar, Aurélie Ponceau, Sophie Vacher, Ivan Bièche, Elisabetta Marangoni, Laetitia Fuhrmann, Anne Vincent-Salomon, Rosette Lidereau, Keltouma Driouch

**Affiliations:** 1 Pharmacogenomics Unit, Genetics Department, Institut Curie, 26 rue d’Ulm, 75248 Paris cedex 05, France; 2 Translational Research Department, Institut Curie, PSL Research University, Paris, France; 3 Pathology Department, Institut Curie, Paris, France

**Keywords:** Kindlins, Redundant functions, Invasion, Breast cancer, Prognosis

## Abstract

**Background:**

Kindlin-1, − 2, and − 3 are the three members of the Kindlin family. They are best known as regulators of integrin functions, contributing to fundamental biological processes such as cell survival, adhesion and migration. Their deregulation leads to diverse pathologies including a broad range of cancers in which both, tumor-promoting and tumor-inhibiting functions have been described.

**Methods:**

To better characterize Kindlins implication in breast cancer, in vitro experiments were performed in a series of cancer cell lines. We first assessed their expression profiles and subcellular distributions. Then, their involvement in breast cancer cell morphology, migration and invasion was verified by examining phenotypic changes induced by the depletion of either isoforms using RNA interference. An expression study was performed in a series of breast cancer patient derived xenografts (*n* = 58) to define the epithelial and stromal contribution of each Kindlin. Finally, we analyzed the expression levels of the three Kindlins in a large series of human breast tumors, at the RNA (*n* = 438) and protein (*n* = 129) levels and we evaluated their correlation with the clinical outcome.

**Results:**

We determined that Kindlin-1 and Kindlin-2, but not Kindlin-3, were expressed in breast tumor cells. We uncovered the compensatory roles of Kindlin-1 and -2 in focal adhesion dynamics and cell motility. Remarkably, Kindlin-2 had a predominant effect on cell spreading and Kindlin-1 on cell invasion. In line with these experimental observations, Kindlin-1 overexpression was associated with a worse patients’ outcome. Notably, Kindlin-3, expressed by tumor infiltrating leukocytes, also correlated with a poor prognosis of breast cancer patients.

**Conclusion:**

This study demonstrates that each one of the Kindlin family members has a different expression profile emphasizing their redundant and complementary roles in breast tumor cells. We highlight the specific link between Kindlin-1 and breast cancer progression. In addition, Kindlin-3 overexpression in the tumor microenvironment is associated with more aggressive breast tumors.

These results suggest that Kindlins play distinctive roles in breast cancer. Kindlins may be useful in identifying breast cancer patients with a worst prognosis and may offer new avenues for therapeutic intervention against cancer progression.

**Electronic supplementary material:**

The online version of this article (10.1186/s13046-018-0955-4) contains supplementary material, which is available to authorized users.

## Background

Breast cancer is the most common malignancy amongst females and one of the leading causes of cancer-related death worldwide. Despite advances in molecular classifications based on gene expression profiles, tumor mutational portraits, and immunochemical markers, patients with metastatic breast cancer still have dismal survival [1–5]. Therefore, identification of novel biomarkers to better assess the prognosis and improve the therapeutic strategies is of great importance.

Kindlins are a family of focal adhesion proteins consisting of three members: Kindlin-1, − 2 and − 3 encoded by *FERMT1*, *FERMT2* and *FERMT3* genes, respectively [6]. Kindlin-2 was the first to be discovered as the mitogen-inducible gene 2 protein [7]. It was shown to be an important component of the integrin-containing focal adhesion structures and indispensable in the proper orchestration of actin assembly and cell shape [8]. The three Kindlins have a high-sequence homology and are known to bind to β-integrins cytoplasmic tails [9] regulating fundamental biological processes such as cell adhesion, spreading, migration, survival, and differentiation [10–13].

Numerous studies have reported Kindlins altered expression levels in a broad range of cancers [14]. Remarkably, both tumor promoting and tumor inhibiting functions of Kindlins have been described dependent on the tumor-type. Kindlin-1 exhibited a tumor suppressor activity in skin tumors whereas it has been shown to promote tumor progression in breast cancer [15–17]. For Kindlin-2, increased levels were reported to enhance tumor invasion and poor prognosis in breast, bladder, pancreas, stomach cancers and malignant mesothelioma, whereas they correlated with reduced tumor invasion and metastasis in colorectal and serous epithelial ovarian cancers [14, 18–23]. Moreover, recent studies provided conflicting results suggesting either a tumor suppressor or a tumor promoting activity of Kindlin-3 in breast cancer and melanomas [14, 24–26].

Many tumor types concomitantly express more than one member of the Kindlin family. In osteosarcomas, Kindlin-1 and -2 up-regulation was associated with a higher tumor grade and a poor prognosis [27], whereas they were found differentially expressed in lung and esophageal cancers where they might oppositely regulate cancer progression [28, 29]. The question of the involvement of the three Kindlins in breast tumors has never been addressed; whether they have redundant and/or complementary roles in mammary tumors remains largely unknown.

In this study, we attempted to discriminate the respective roles of Kindlins on cell morphology and the migration and invasion capacities of breast cancer cells. We also performed an integrated expression analysis of all three transcripts and proteins in large series of different breast tumor subtypes and patients-derived xenografts. We precisely determined the epithelial versus stromal origin of Kindlins expression in breast tumors. Finally, we evaluated their prognosis value for patient’s outcome.

## Materials and methods

### Cell culture and Kindlins transient knockdown

Human cell lines MCF7, ZR-75-1, SKBR3, BT-20, MDA-MB-453, MDA-MB-231, MDA-MB-468, Hs.578 T and THP1 were purchased from ATCC (Manassas, VA, USA), maintained at 37 °C with 5% CO_2_ and grown in DMEM, MEM or RPMI 1640 medium supplemented with 10% FBS and 1% antibiotics (50 μg/mL penicillin, 50 μg/mL streptomycin, 100 μg/mL neomycin).

Transfections were performed using Lipofectamine (Invitrogen, Carlsbad, CA, USA) following the manufacturer’s protocol with siRNA-negative control (D-001210-03) or siRNA-Kindlin-1 (D-004511-02) and/or siRNA-Kindlin-2 (D-012753-01) from Dharmacon (Lafayette, CO, USA).

### Western Blotting and immunofluorescence

For western blotting, cells were lysed using RIPA buffer (50 mMTris–HCl, pH 8; 150 mMNaCl; 0.5% triton; 0.5% deoxycholic acid) containing protease inhibitors (1:100 orthovanadate, 1:100 apoprotinine, 1:200 PMSF). Protein extracts were loaded on a polyacrylamide gel, transferred to a nitrocellulose membrane and incubated, overnight at 4 °C, with primary antibodies for Kindlin-1 (1:10000, [16]; Kindlin-2 (1:5000, Clone3A3, Millipore, Billerica, MA); or Kindlin-3 (1:1000, D817V, Cell signaling, Danvers, MA). GAPDH was used as loading control (1:10000, Clone V18, Santa Cruz Biotechnologiy, Santa Cruz, CA). The signals were detected according to the ECL Western Blotting Analysis System procedure (GE Healthcare, Buckinghamshire, UK).

For immunofluorescence, transfected cells were fixed in 4% paraformaldehyde, permeabilized and immunostained with primary antibodies (anti-Kindlin-1 [16], 1:700; anti-Kindlin-2, clone 3A3, 1:2000) followed by alexa fluor-conjugated secondary antibodies (A11031, and A11034, Invitrogen). Cells were then counterstained with DAPI and imaged with the fluorescence Eclipse Ti microscope from Nikon (Melville, NY, USA).

### Time-lapse migration assay

Migration assays were conducted on an Eclipse Ti-E inverted full-motorized microscope (Nikon) equipped with an incubation chamber (OKOlab, Pozzuoli, Italy) maintained at 37 °C with 5% CO_2_. Movies were acquired by an ORCA Flash 4.0 V2 digital CMOS camera (EPI light path, Hamamatsu Photonics, Japan) controlled by NIS-Elements BR 3.0 software (Nikon). Cell migration was recorded for 24 h. Single cells’ tracking was conducted using the “Manual Tracking” plugin of ImageJ software (NIH, Bethesda, MA, USA).

### Transwell invasion assay

Invasion assays were performed using inserts with 8.0 μm pore size membranes according to the manufacturer’s protocol (Becton Dickinson, Franklin Lakes, NJ, USA). The bottom side of the chamber was pre-coated with 4 μg/cm^2^ of Matrigel (BD Biosciences, San Jose, CA, USA) and 10% FBS culture medium was used as chemoattractant in the lower chamber. 2.10^4^ cells were plated in the top chamber. 24 h later, they were fixed, stained with DAPI, imaged with the fluorescence Eclipse Ti microscope (Nikon) and counted to estimate the number of invasive cells.

### Human breast tumors and patient-derived xenografts

The study was performed in accordance with the French Bioethics Law 2004–800 and the French National Institute of Cancer (INCa) Ethics Charter, after approval by the Institut Curie review board and ethics committee.

Total RNA was extracted from 438 primary breast tumor samples collected from patients undergoing surgery at the Institut Curie-Huguenin Hospital. Samples encompass the various stages of breast cancer progression and the molecular subtypes, as previously defined (Table 1) [30]. This cohort consisted of 169 metastasizing tumors. 33 patients relapsed only to the lungs and 60 to the bones within the first 150 months.

**Table 1:** Kindlins expression and correlation with the breast tumors clinicopathological parameters

Clinicopathological variables	No (%)	No. of patients (%)								
		Kind1 expression			Kind2 expression			Kind3 expression		
		Low	High	*p* value	Low	High	*p* value	Low	High	*p* value
Total	438	377 (86.1)	61 (13.9)		364 (83.1)	74 (16.9)		165 (37.7)	273 (62.3)	
SBR histological grade										
I	55	52 (94.5)	3 (5.5)		41 (74.5)	14 (25.5)		23 (41.8)	32 (58.2)	
II	222	202 (91.0)	20 (9.0)		182 (82.0)	40 (18.0)		90 (40.5)	132 (59.5)	
III	153	118 (77.1)	35 (22.9)	0.0002	134 (87.6)	19 (12.4)	NS	49 (32.0)	104 (68.0)	NS
Lymph node status										
0	115	93 (80.9)	22 (19.1)		99 (86.1)	16 (13.9)		48 (41.7)	67 (58.3)	
1–3	229	201 (87.8)	28 (12.2)		190 (83.0)	39 (17.0)		86 (37.6)	143 (62.4)	
> 3	93	82 (88.2)	11 (11.8)	NS	75 (80.6)	18 (19.4)	NS	31 (33.3)	62 (66.7)	NS
Macroscopic tumor size										
≤ 25 mm	214	185 (86.4)	29 (13.6)		174 (81.3)	40 (18.7)		81 (37.9)	133 (62.1)	
> 25 mm	216	185 (85.6)	31 (14.4)	NS	185 (51.5)	31 (43.7)	NS	80 (37.0)	136 (63.0)	NS
ER status										
Negative	111	67 (60.4)	44 (39.6)		91 (82.0)	20 (18.0)		32 (28.8)	79 (71.2)	
Positive	327	310 (94.8)	17 (5.2)	*p* < 0.0000001	273 (83.5)	54 (16.5)	NS	133 (40.7)	194 (59.3)	0.03
PR status										
Negative	184	139 (75.5)	45 (24.5)		153 (83.2)	31 (16.8)		61 (33.2)	123 (66.8)	
Positive	254	238 (93.7)	16 (6.3)	p < 0.0000001	211 (83.1)	43 (16.9)	NS	104 (40.9)	150 (59.1)	NS
HER2 status										
Negative	345	295 (85.5)	50 (14.5)		288 (83.5)	57 (16.5)		137 (39.7)	208 (60.3)	
Positive	93	82 (88.2)	11 (11.8)	NS	76 (81.7)	17 (18.3)	NS	28 (30.1)	65 (69.9)	NS
Molecular subtypes										
ER- PR- HER2- (Triple Neg)	64	29 (45.3)	35 (54.7)		54 (84.4)	10 (15.6)		18 (28.1)	46 (71.9)	
ER- PR- HER2+ (ERBB2)	42	34 (81.0)	8 (19.0)		32 (76.2)	10 (23.8)		9 (21.4)	33 (78.6)	
ER+ PR+ HER2- Ki67^low^ (Lum A)	213	202 (94.8)	11 (5.2)		173 (81.2)	40 (18.8)		98 (46.0)	115 (54.0)	
ER+ PR+ HER2+ or Ki67^high^ (Lum B)	119	112 (94.1)	7 (5.9)	p < 0.0000001	105 (88.2)	14 (11.8)	NS	40 (33.6)	79 (66.4)	0.003

Statistical analyses were performed by means of a chi-squared test (*NS*: not significant)

Kindlins expression analyses were validated using the METABRIC data set (*n* = 2509) [31] publicly available from cBioPortal (www.cbioportal.org/).

For protein expression studies, tissue microarrays (TMAs) consisting of 129 breast tumors and adjacent normal breast tissues from patients treated at the Institut Curie were obtained from the Pathology Department of Hospital Curie (Additional file 1: Table S1).

Fifty eight breast cancer patient-derived xenograft models (Additional file 1: Table S2) were also obtained from the Laboratoire d’Investigation Pré-clinique (Institut Curie) as previously described [32].

### Immunohistochemistry

TMAs were deparaffinized in toluene, rehydrated in ethanol and H_2_O, submerged in Tris-EDTA retrieval buffer (10 mM Tris-base, 1 mM EDTA, 0.05% Tween 20, pH 9), treated with peroxidase blocking reagent (Dako, Ely, UK) and incubated at 4 °C overnight with primary antibodies(anti-kindlin-1, 1:500 AB68041, Abcam, Cambridge, MA; anti-Kindlin-2, 1:100 Clone 3A3; anti-Kindlin-3, 1:400 D817V). The staining signals were revealed with the Dako REAL Detection System, Peroxidase/AEC kit (Dako). Slides were counterstained with Mayer’s hematoxylin. For the semi-quantitative analysis, the H-score method assigned a score of 0–300 to each patient, based on the percentage of cells stained at different intensities.

### Quantitative real-time polymerase chain reaction (qRT-PCR)

Total RNA extraction, cDNA synthesis, and normalization methods were described elsewhere [33]. Transcripts levels were calculated using the ΔΔ*C*t method and normalized to the TBP mRNA levels. The primers’ sequences are listed in Supplementary Table S3 (Additional file 1).

### Statistical analysis

Statistical calculations were performed using PASW Statistics (version 18.0; SPSS Inc.). The optimal cutoff point to categorize patients into high versus low Kindlins expression groups was determined by use of the receiver operating characteristic method. Survival distributions were estimated by the Kaplan Meier method, and the significance between survival rates was ascertained using the log-rank test. Multivariate analysis using Cox proportional hazards model was used to assess the independent contribution of each variable to metastasis-free survival.

## Results

### Kindlins expression in breast cancer cells

**Fig. 1:**
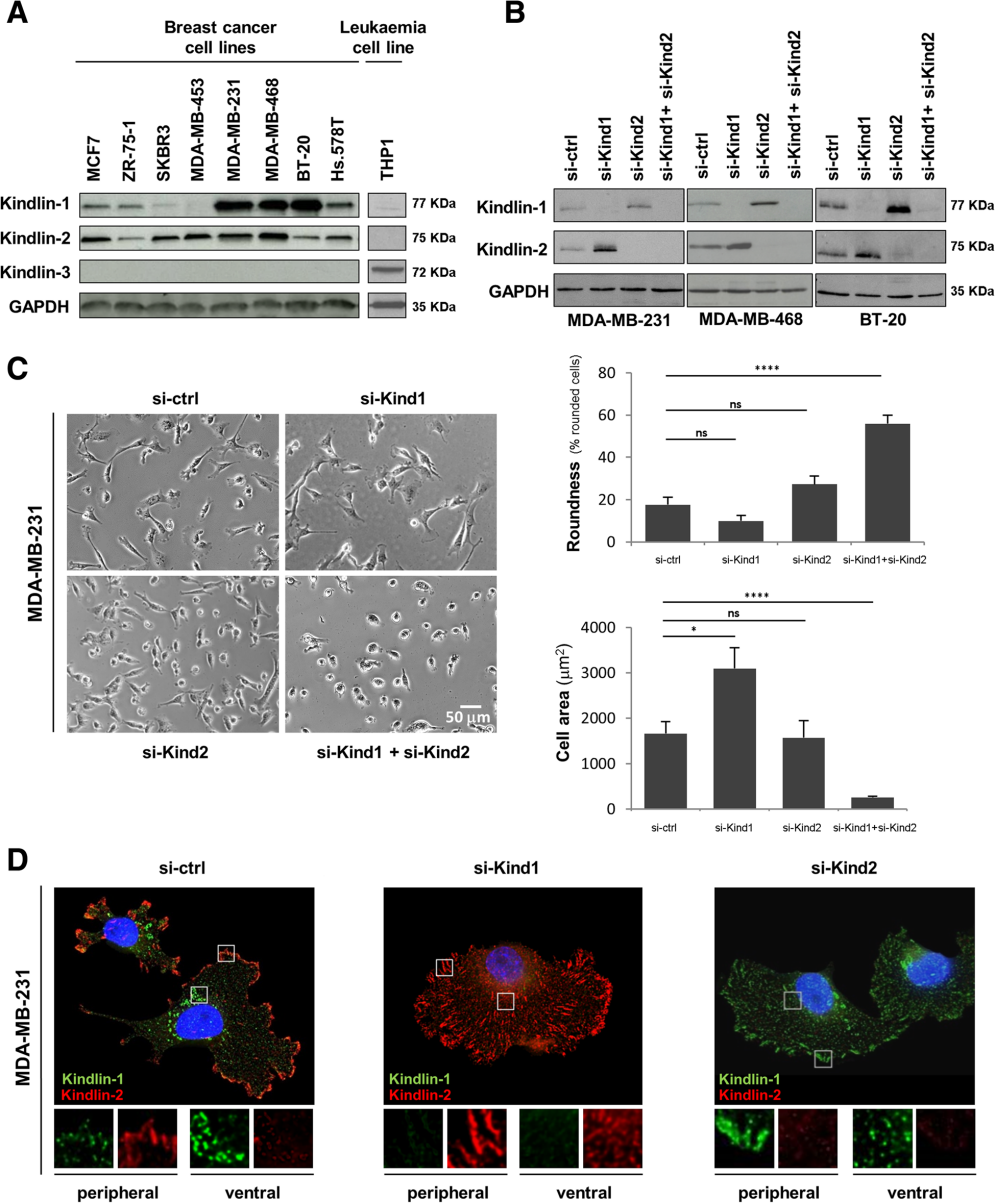
Kindlin-1, −2 and − 3 expression, involvement in morphology and subcellular localization in breast cancer cells. **a** Western Blots were performed in order to compare protein levels of Kindlin-1, − 2 and − 3 between different breast cancer cell lines and a hematopoietic cell line (THP1). **(b-d)** MD-MB-231, BT20 and MDA MB 468 cells were transfected with control siRNA (si-ctrl), *KIND1* siRNA (si-Kind1), *KIND2* siRNA (si-Kind2) alone or in combination (si-Kind1 + si-Kind2) for five days. **b** Cellular extracts were immunoblotted with anti-Kindlin-1, anti-Kindlin-2, and anti-GAPDH (loading control) antibodies. **c** Phase contrast microscopy was performed in MDA-MB-231 cells to calculate the roundness (% of rounded cells) and cell area (μm^2^) represented as the mean +/− SEM of values. Statistical analysis were made by performing a *t-test* (****p < 0.0001; ***p < 0.001; *p < 0.05; ns: not significant). **d** Five days after transfection, MDA-MB-231 cells were also fixed, permeabilized and immunostained with anti-Kindlin-1 and anti-Kindlin-2 antibodies. Cells were then counterstained with DAPI and imaged with a fluorescence microscope (original magnification: X100)

To better characterize the role of Kindlins in breast cancer, we first examined their protein expression in several cancer cell lines. We found that most breast tumor cells expressed both Kindlin-1 and Kindlin-2, whereas Kindlin-3 was only detected in the human monocytic THP1 cells, consistent with previous studies reporting that Kindlin-3 expression is restricted to hematopoietic cells [12, 34] (Fig. 1a). Among the breast cancer cell lines, Kindlin-2 levels showed only slight variations as compared to Kindlin-1 which varied from not detectable to highly expressed (Fig. 1a).

To discriminate Kindlin-1 and -2 functions in vitro, we investigated the impact of their depletion on breast cancer cells. We selected those cells with highest levels of both Kindlin-1 and -2 proteins. MDA-MB-231, MDA-MB-468, BT-20 and Hs.578 T cells were thus knockdown for Kindlin-1 and/or Kindlin-2. Immunoblots demonstrated efficient knockdown, both in single and double silencing conditions (> 95%, Fig. 1b and Additional file 2: Figure S2A). Importantly, Kindlin-1 depleted cells exhibited an increased Kindlin-2 expression and vice versa (Fig. 1b, and Additional file 2: Figure S2A) in all four tested lines. These results suggested that kindlin-1 and Kindlin-2 expression may compensate for each other in breast cancer and the switch was unlikely due to a cell line-specific process.

### Kindlin-1 and Kindlin-2 control breast cancer cell shape and size

We then examined the morphology of Kindlins-depleted cells by phase contrast microscopy (Fig. 1c and Additional file 2: Figure S1A). Concomitant silencing of both Kindlins had a drastic effect on breast cancer cells which completely lost their initial morphology becoming rounded. Double-depleted MDA-MB-231 cells showed a 3-fold increase of the number of round cells as compared to control cells (*p = 4.10*^*− 6*^, Fig.1c). In addition, these cells were significantly smaller (si-ctrl: 1662 ± 267 μm^2^; si-Kind1 + si-Kind-2: 261 ± 28 μm^2^, *p = 10*^*6*^, Fig. 1c) and massively detached from the plate.

In contrast, depletion of either Kindlin-1 or Kindlin-2 alone had a limited or no effect on the number of rounded cells and cell size suggesting that they could compensate for each other to maintain cell morphology (Fig. 1c and Additional file 2: Figure S1A).

Noteworthy, instead of the decrease in cell size expected for Kindlin-1 depleted cells, we observed a significant increase in cell spreading. This observation strongly suggested that cell spreading, not relying any longer on Kindlin-1, was over-compensated by the up regulation of Kindlin-2 (Fig. 1c). This effect was not observed in Kindlin-2 depleted cells despite the up-regulation of Kindlin-1, suggesting a weaker involvement of Kindlin-1 in cell spreading.

### Kindlin-1 and Kindlin-2 subcellular localization in breast cancer cells

To better understand the distinct and redundant functions of Kindlins in breast cancer cells, we investigated their subcellular localization by immunofluorescence. In control MDA-MB-231 cells, Kindlin-1 showed a dot-like staining predominantly at the perinuclear region. This pattern was reminiscent of Kindlin-1 localization at the ventral adhesions reported in keratinocytes [35]. Kindlin-2 staining appeared as large patches at the cell periphery, indicating that it resides mainly in the peripheral focal adhesions (Fig. 1d and Additional file 2: Figure S1B). Thus, in breast cancer cells Kindlin-1 and -2 can localize to different adhesion sites. Of note, the BT-20 cell line showed a more heterogeneous staining with a majority of cells expressing only one Kindlin (Additional file 2: Figure S1B).

When silencing Kindlin-1, cells presented an increased number of Kindlin-2-positive focal adhesions at the periphery, consistent with the up-regulation of Kindlin-2 observed by western blot (Fig.1d and b). Interestingly, following Kindlin-2 silencing, an extended relocation of Kindlin-1 from ventral to peripheral adhesions was observed (Fig. 1d and Additional file 2: Figure S1B).

These results suggested a different localization of Kindlin-1 and Kindlin-2 in breast cancer cells. However, Kindlin-1 may relocate upon the inactivation of Kindlin-2 likely to compensate its functions in vitro.

### Kindlins requirement for breast cancer cell migration and invasion

**Fig. 2:**
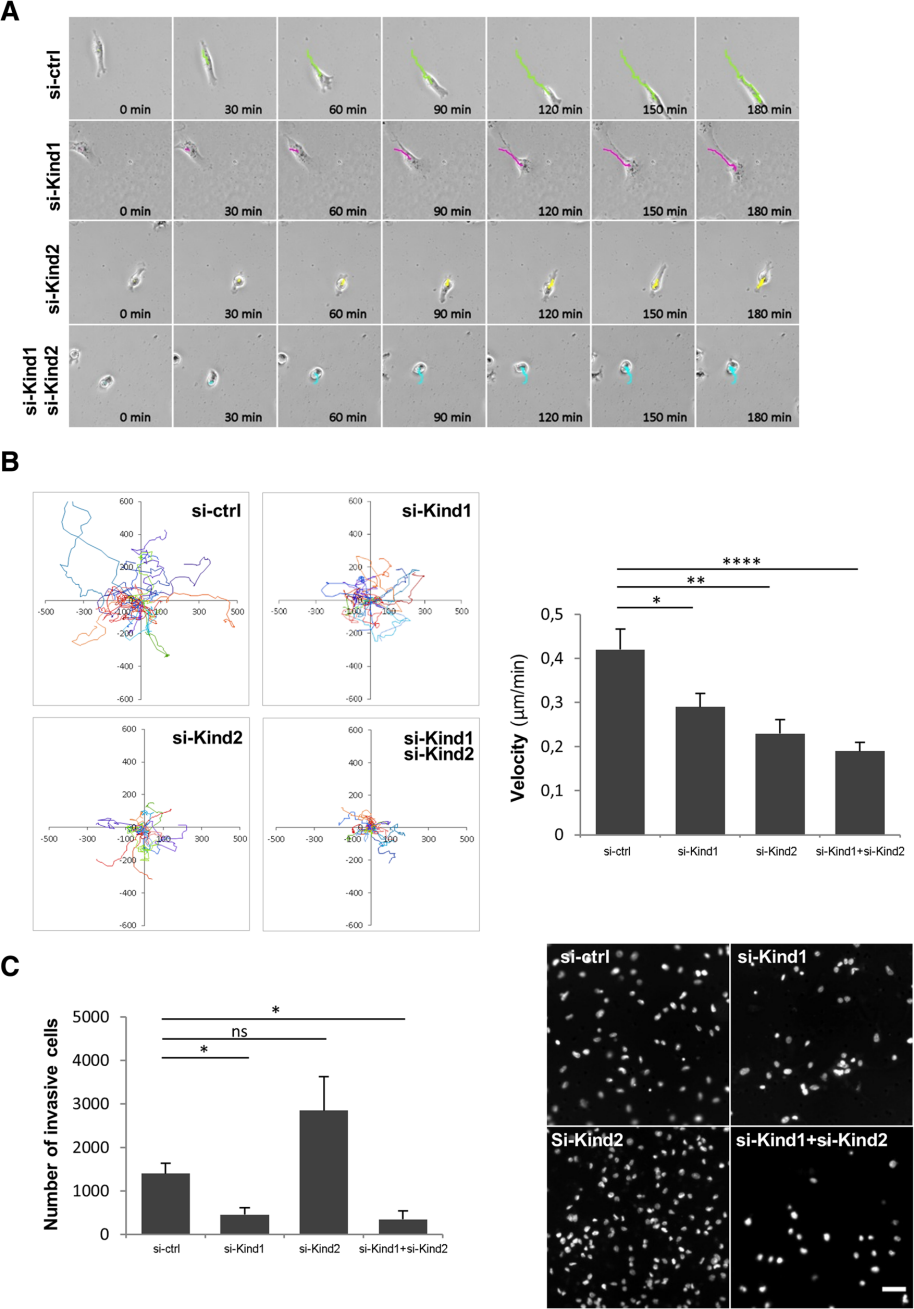
Kindlin-1 and Kindlin-2 involvement in breast cancer cell motility. MD-MB-231 cells were transfected with control siRNA (si-ctrl), *KIND1* siRNA (si-Kind1), *KIND2* siRNA (si-Kind2) alone or in combination (si-Kind1 + si-Kind2) for five days. **a** Time-lapse imaging was performed for 24 h. Images show representative trajectories travelled by cells during 180 min. **b** Plots show overlays of the representative trajectories travelled by cells .Velocity was quantified and represented as the mean +/− SEM of values (*n* = 20 cells tracked by condition). Statistical analysis were performed by a *t-test* (****p < 0.0001; **p < 0.01; *p < 0.05). Results are representative of experiments performed at least in duplicate. **c** A transwell cell invasion assay was performed for 24 h. Cells were then counterstained with DAPI and imaged with a fluorescence microscope. The number of invasive cells was quantified and represented as the mean ± SEM of values. Statistical analyses were performed by a *t-test* (*p < 0.05; ns: not significant). A representative image of three independent experiments is shown for each condition. Scale bar = 50 μm

We then evaluated Kindlins role in breast cancer cell motility. We first tested cell migration of MDA-MB-231 and Hs.578 T cells knockdown for Kindlin-1 and/or Kindlin-2 by a time-lapse microscopy experiment. Figure 2a and b showed that cell migration was significantly impaired in MDA-MB-231 cells when silencing both Kindlins; we observed a two-fold decrease in cell velocity as compared to control cells (0.19 μm/min vs. 0.42 μm/min, respectively, *p = 10*^*− 4*^). By silencing only one Kindlin, cells migrated slower than si-ctrl cells with a slightly higher effect of si-Kind2 (*p = 10*^*− 3*^). Similar results were obtained in Hs.578 T cells (Additional file 2: Figure S2B).Once again, these results suggested a redundancy in Kindlin’s function in breast cancer cells.

We then analyzed the invasive capacities of these cells. Same as for migration, silencing of both Kindlins induced an important decreased in the number of invasive cells both in MDA-MB-231 (si-ctrl: 1401 ± 243 cells; si-Kind1 + si-Kind2:349 ± 196 cells, *p = 0.02*, Fig. 2c) and Hs.578 T cells (ctrl: 2545 ± 401 cells; si-Kind1 + si-Kind2: 818 ± 446, *p = 0.007*, Additional file 2: Figure S2C). When performing single silencing, si-Kind1-treated cells exhibited a significant decrease of the MDA-MB-231 (si-Kind1: 460 ± 155 cells, *p = 0.02*) and Hs.578 T (si-Kind1: 1225 ± 322 cells, *p = 0.04*) cells invasive capacities. In contrast, Kind2-depleted cells did not exhibit a difference in cell invasion as compared to control cells (si-Kind2: 2846 ± 782 cells, *p = 0.13*); even though a slight decrease was observed for Hs.578 T cells (Additional file 2: Figure S2C). Our results suggested that Kindlin-1 has a more prominent role than Kindlin-2 in breast cancer cell invasion.

Altogether, our findings suggested that Kindlin-1 and kindlin-2 have the ability to partially compensate each other in breast cancer cells. We determined an important role of Kindlin-1 and Kindlin-2 in controlling cell shape, adhesion and migration. Our results also indicated a higher involvement of Kindlin-2 in cell spreading and a more important role of Kindlin-1 in cell invasion. 

### Kindlins cell-specificity in human breast tissues

Our observations prompted us to investigate the putative redundant and/or distinct functions of Kindlins in human breast tumors. Although we determined that Kindlin-3 was not detected in breast cancer cell lines, we wondered whether it could be expressed de novo in human tumor cells and whether its stromal expression could affect breast cancer progression as previously suggested [26].

**Fig. 3:**
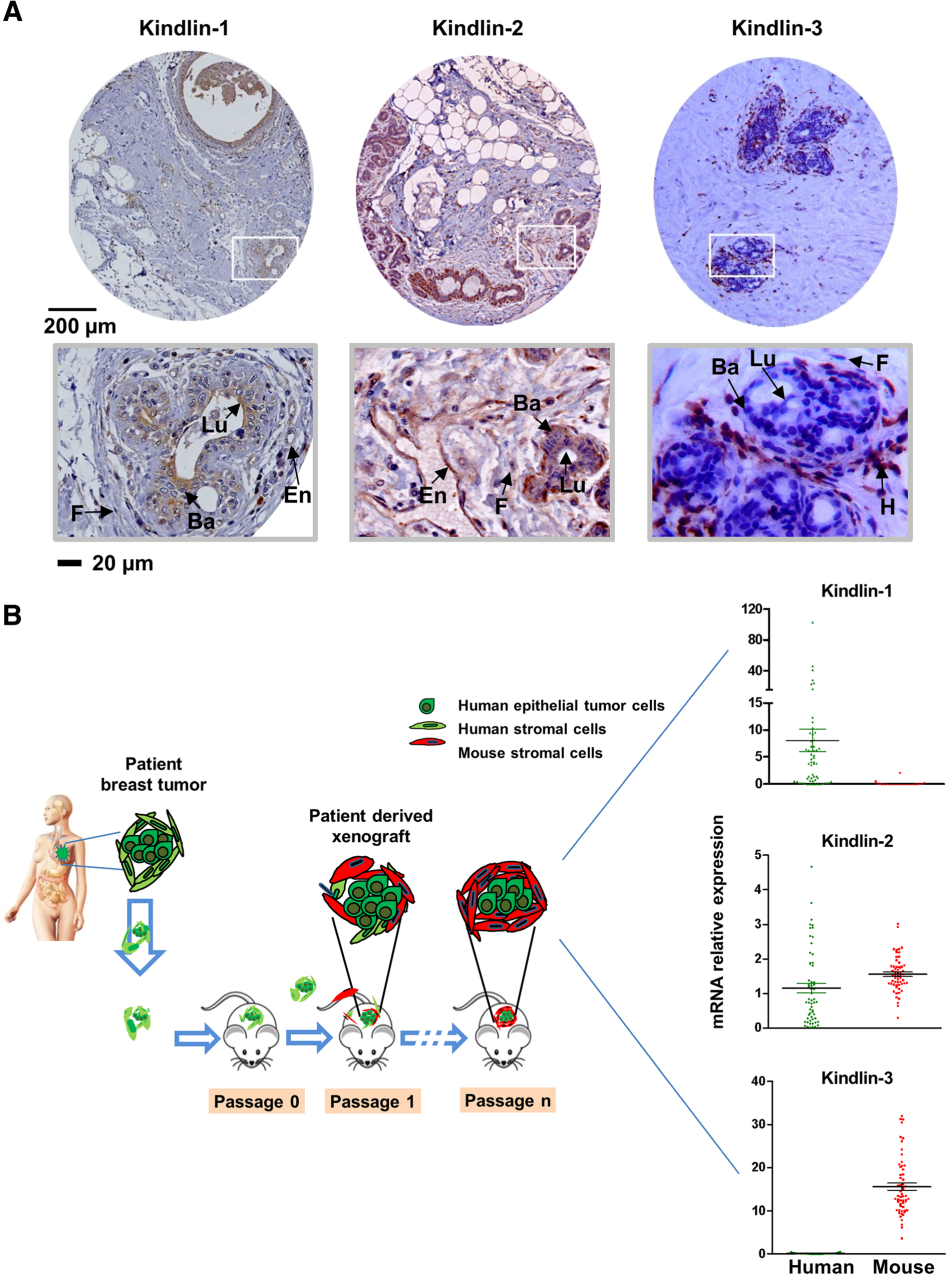
Kindlins expression is cell-type specific. **a** Immunohistochemical staining of the normal mammary gland were performed in different patient’s samples to analyze the levels and localizations of these proteins (En: endothelial cells, F: fibroblasts, H: hematopoietic cells, Lu: luminal epithelial cells, Ba: basal epithelial cells). **b** Establishment of breast cancer patient-derived xenografts (PDX): Primary breast tumor fragments derived from patients are engrafted into immunocompromised mice. Tumors can be implanted into the interscapular fat pad, the mammary fat pad or in the flank. Xenografts appear at the graft site 1–12 months after grafting, they are subsequently transplanted from mouse to mouse; adapted from [44]. Then, Kindlins transcript levels were assessed in bulk tumors by using species-specific primers (mean ± SE values are represented)

We first assessed Kindlins expression in normal mammary glands, performing an immunohistochemical analysis. As expected, Kindlin-1 protein was predominantly localized in epithelial cells (negative to moderate expression) while Kindlin-2 was detected not only in epithelial cells (moderate to high expression) but also in fibroblasts and endothelial cells. Kindlin-3 localization was restricted to the infiltrating immune cells (Fig. 3a).

To better define the stromal contribution of Kindlins expression in breast tumors, we analyzed a series of 58 breast cancer patients’ derived xenografts (PDX) by means of real-time qRT-PCR. As depicted in Fig. 3b, over the passages, human stromal cells were substituted by mouse stromal cells. Thus, bulk PDX tumors were finally composed of human epithelial tumor cells surrounded by mouse stromal microenvironment (Fig. 3b). By using species-specific primers, we could demonstrate that Kindlin-1 was exclusively amplified in human epithelial cancer cells. Kindlin-2 was detected with both human and mouse primers indicating an epithelial and stromal expression. Finally, Kindlin-3 was exclusively amplified in mouse cells, suggesting an exclusive expression in the tumor microenvironment (Fig. 3b).

### Kindlins differential expression in breast tumors

**Fig. 4:**
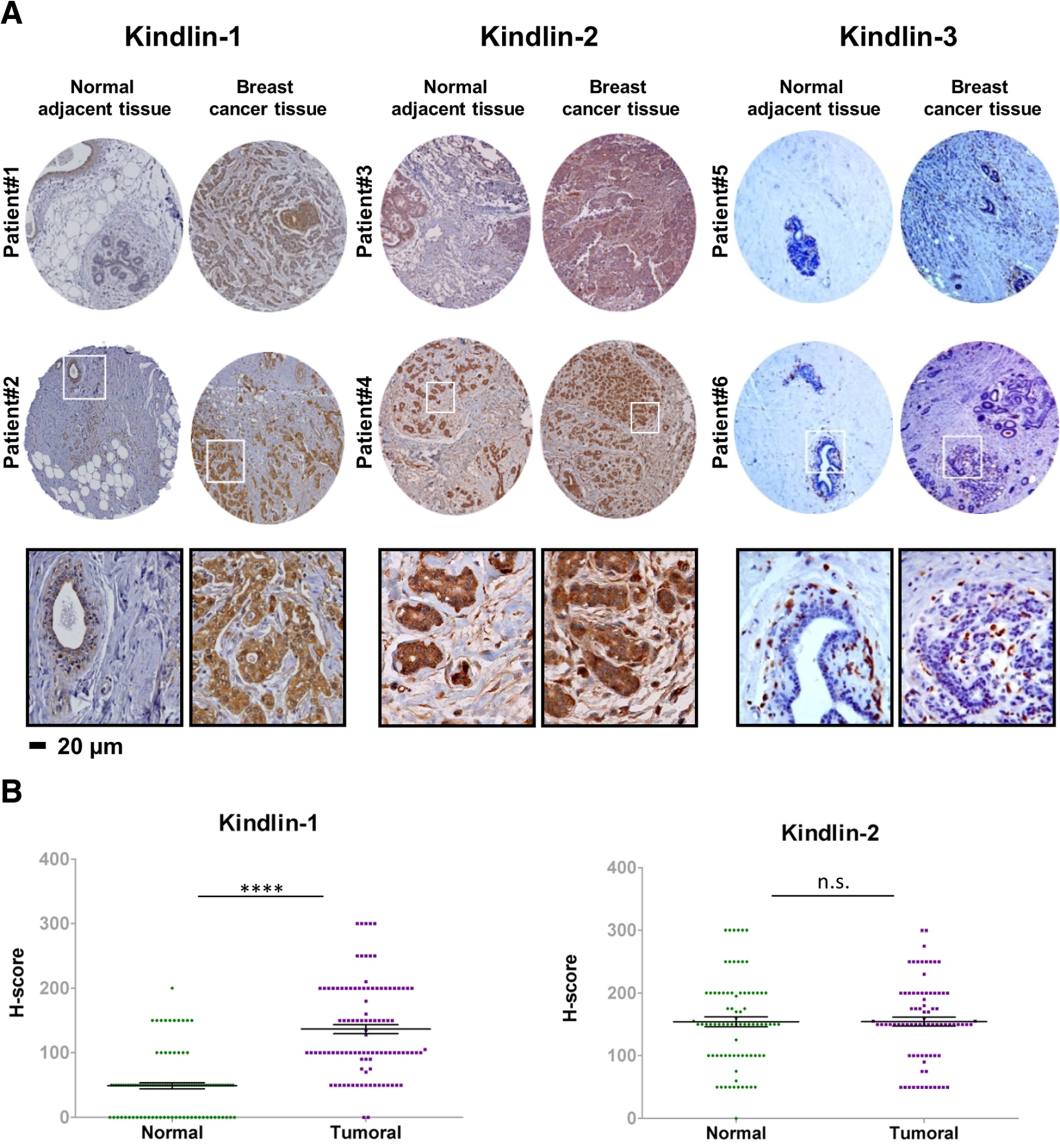
Kindlin-1, − 2 and − 3 protein expressions in breast cancer and adjacent noncancerous tissue. **a** Representative images of Kindlin-1, − 2 and − 3 immunohistochemical staining in breast cancer TMA and normal adjacent tissues. **b** Scatter plot showing range of Kindlin-1 and -2 expressions related to histology. Each point represents the H-score from a single tissue sample ranging from total absence of Kindlin in the epithelial compartment (H-score 0), to very strong Kindlin staining (H-score 300). Mean H-score ± SE represented. Statistical differences between normal and tumor expression levels were performed using the Mann-Whitney *test*

We next performed an immunohistochemical analysis to evaluate Kindlins expression in a TMA consisting of 129 human breast tumors. We compared the expression of the different Kindlins in the tumors and the normal adjacent tissues of the same patients (Fig. 4a). First, consistent with breast cancer cell lines, none of the tested tumors showed Kindlin-3 expression in epithelial cells. The intensity levels of Kindlin-1 and -2 staining were plotted using the H-score (Fig. 4b). Kindlin-1 protein levels showed a significant increase in the tumor tissues vs. normal adjacent tissues (*p < 10*^*− 4*^) suggesting a potential role of Kindlin-1 in breast cancer. In contrast, Kindlin-2 exhibited a wide range of expression with no significant differences between the normal and tumoral breast tissues (Fig. 4b).

**Fig. 5:**
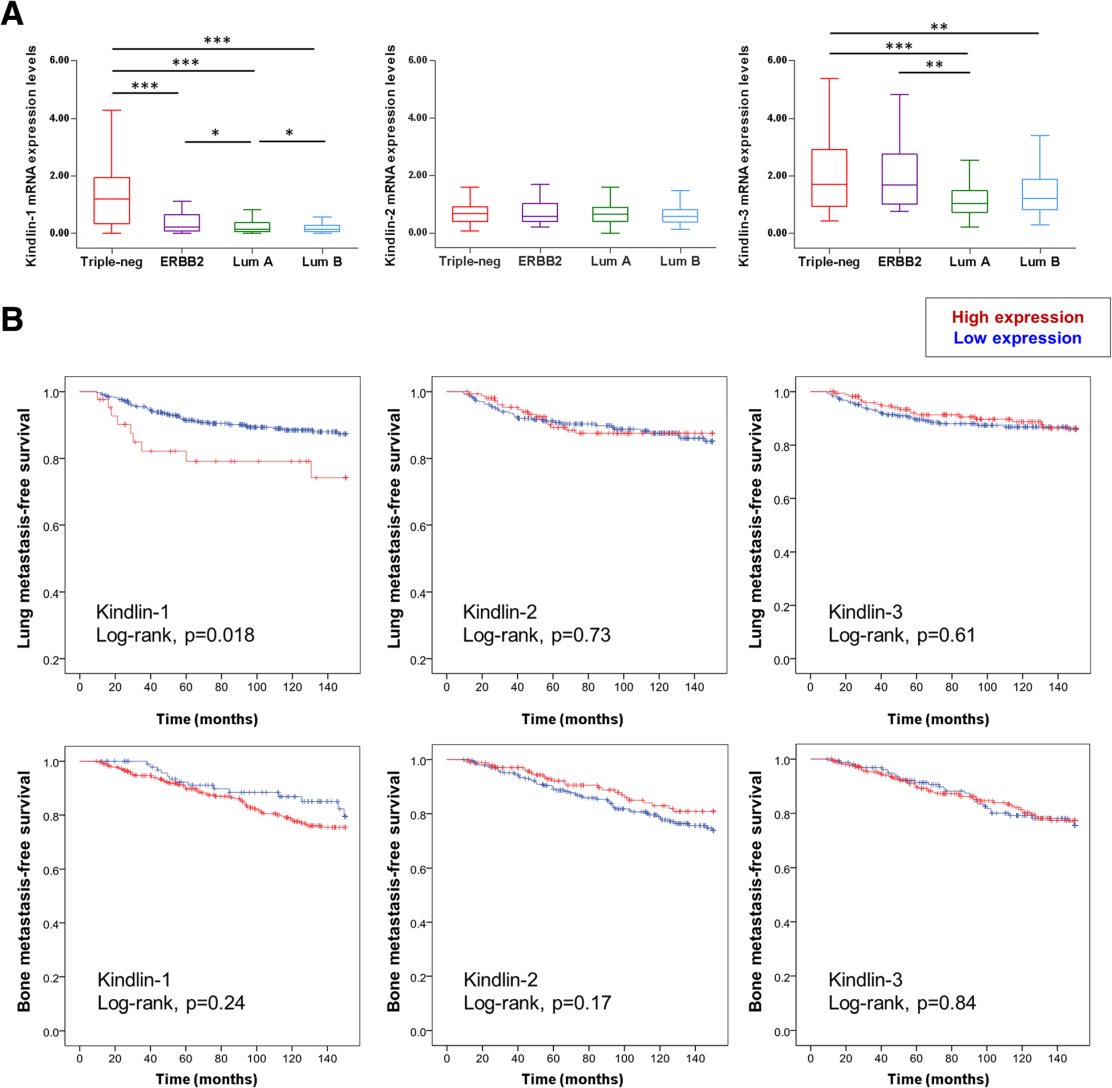
Kindlin-1 expression correlates with lung metastasis. **a** Box-and-Whisker plot showing kindlin-1, − 2 and − 3 mRNA expression levels in a series of 438 breast cancer patients grouped according to four well described breast tumor molecular subtypes (Triple negative, ERBB2, Luminal A and Luminal B). Statistical analysis were made by performing Tukey’s multiple comparison *test* (***p < 0.001; **p < 0.01; *p < 0.05). **b** Kaplan-Meier curves showing lung and bone metastasis-free survival of patients with tumors expressing high (red lines) vs. low (blue lines) levels of Kindlin-1, − 2 and − 3 analyzed by qRT-PCR. Statistical analyses were performed by a Log-rank test

To further determine whether Kindlins’ expression was associated with breast cancer progression, we analyzed Kindlins transcripts in a large series of 438 breast cancer patients (Table 1). We examined Kindlins expression with regard to different clinicopathological parameters. We found that tumors expressing high levels of Kindlin-1 were more frequently of an advanced grade (grade III) and they showed more often hormone receptors (ER and PR) negativity (*p = 2.10*^*− 4*^*, p < 10*^*− 7*^and *p < 10*^*− 7*^respectively). Kindlin-1 expression also showed an unbalanced distribution according to the different molecular subtypes of breast tumors (Table 1). The triple-negative subgroup, the one with the worst survival rates, had the highest levels of *Kindlin-1* transcripts (*p < 10*^*− 3*^, Fig. 5a) confirming our previously reported results [16].

In contrast, Kindlin-2 was not associated with any of the clinicopathological parameters (Table 1 and Fig. 5a). Of note, although expressed by stromal cells, increased levels of Kindlin-3 were found to correlate with the ER status and the molecular subtype (*p = 0.03* and *p = 0.003* respectively). The highest Kindlin-3 expression levels were observed in triple negative and ERBB2 tumors (Table 1 and Fig. 5a). To determine whether Kindlin-3 expression may reflect the amount of tumor infiltrating leukocytes (TILs) we analyzed the mRNA levels of different immune markers in the same series of tumors and found that triple negative and ERBB2 tumors presented the highest levels of CD45, CD86, CD28 and CD4 (Additional file 2: Figure S3A). Furthermore, Kindlin-3 expression highly correlated with the levels of these immune markers (*p < 10*^*−7*^*,* Additional file 2: Figure S3B). To validate our findings, we examined Kindlins’ expression among an independent series of breast tumors using publicly available data from the METABRIC project. We obtained similar results for all three Kindlins (Additional file 2: Figure S4).

### Kindlins expression and patients’ outcome

We then evaluated the prognostic value of Kindlins in breast cancer. First, we analyzed the impact of Kindlins expression on the metastasis-free survival of Curie breast cancer patients (Additional file 2: Figure S5A). We observed that Kindlin-3 showed a tendency towards a poor prognosis (*p = 0.056*). Moreover, when we analyzed the impact of Kindlins in the METABRIC cohort we observed that Kindlin-3 expression was highly associated with a decreased overall survival in this larger series (Additional file 2: Figure S5B, *p = 3.10*^*− 5*^) and Kindlin-1 tended to be associated with a poor prognosis (*p = 0.058* and *p = 3.10*^*− 5*^ at 120 and 60 months, respectively).

Consistent with previous findings [16, 36, 37], in our series of 438 patients, Kindlin-1 showed a higher expression in lung metastizing tumors if compared with bone metastizing tumors (*p < 0.001).* Importantly, Kindlin-2 and -3 expressions did not differ according to the metastatic site (Additional file 2: Figure S6). We thus evaluated the correlation between Kindlins expression and lung and bone metastasis-free survival rates (Fig. 5b) corroborating that only a high Kindlin-1 expression was associated with a poor patient’s outcome regarding to lung metastasis (*p = 0.018*). Moreover, a multivariate analysis showed that the involvement of Kindlin-1 in lung metastasis was independent of the other clinicopathological parameters (Table 2).

**Table 2:** Correlation of Kindlin-1 expression with lung metastasis (*n* = 438)

Parameter		Univariate analysis			Multivariate analysis		
		*p* value	HR	95%CI	*p* value	HR	95%CI
Kindlin-1 expression	Low	0.022	1		0.017	1	
	High		2.330	1.132–4.795		2.412	1.169–4.977
Lymph node status	0–3	0.042	1		0.067	1	
	> 3		1.878	1.024–3.443		1.784	0.960–3.314
Macroscopic tumor size	≤ 25 mm	0.032	1		0.078	1	
	> 25 mm						
			1.872	1.057–3.315		1.688	0.942–3.025
SBR histological grade	I-II	0.161	1				
	III		1.507	0.849–2.677			
Age	≤ 55	0.946	1				
	> 55		1.021	0.563–1.850			

*HR*: hazard ratio, *CI*: confidence intervals

## Discussion

Over the past few years, several reports have emphasized Kindlins either as positive or negative regulators of cancer cell metastasis. Our present work aimed at clarifying Kindlins expression and their respective roles in breast cancer. To our knowledge, this is the first study to report an integrated analysis of the three Kindlin members in breast cancer.

First, we demonstrated that Kindlin-1 and Kindlin-2 were concomitantly expressed in several breast tumor cell lines. Importantly, inactivation of one of these proteins led to the up-regulation of the other one, and vice versa, suggesting that a relative level of Kindlin expression is required for cell homeostasis. Of note, in fibroblasts, loss of Kindlin-2 was shown to be compensated by de novo expression of Kindlin-1 [38]. However, this expression switch was not observed in keratinocytes [35, 39–41] suggesting that this process might depend on the type of tissue or cellular conditions.

We also demonstrated that simultaneous loss of Kindlin-1 and Kindlin-2, drastically impacted breast cancer cell shape, cell size and cell migration, as reported in keratinocytes and fibroblasts [38, 41]. In contrast, we showed that loss of either of these proteins had a limited effect highlighting their redundant functions and their ability to compensate each other, in line with previous works [41].

Despite their similarities, Kindlin-1 and -2 also exhibited unique functions [39]. Consistent with other groups, we observed a stronger involvement of Kindlin-2 in focal adhesion formation and cell spreading [39, 41]. Similarly, Kindlin-1 has a specific role in cell adhesion to fibronectin-rich extracellular matrix (EMC) [39, 41]. In a mouse model deficient for Kindlin-1, Kindlin-2 expression was unable to compensate the capacity of colonic epithelial cells to adhere to the basal membrane confirming this unique function of Kindlin-1 [42]. At the molecular level, Kindlins specificities were demonstrated to rely on their respective affinity for β-integrins. For example, Kindlin-1 but not Kindlin-2 is able to bind to β6-integrins [39, 42].

Strikingly, we provided evidence that Kindlin-1 rather than Kindlin-2 expression was required to drive breast cancer cell invasion. In line with our in vitro experiments, we found that only Kindlin-1 was overexpressed in human breast tumors. We also provided clinical evidence for the involvement of Kindlin-1 overexpression in breast cancer progression, corroborating our previous works, at the protein level and in larger data sets [16, 37]. In agreement with our observations in breast cancer, other groups have also demonstrated the role of Kindlin-1 in cell invasion in distinct cancer types including pancreatic and colorectal carcinomas. In these cancers, the up-regulation of Kindlin-1 was also correlated with cancer progression and poor patient outcomes [43, 44].

By contrast, in breast cancer, we found that Kindlin-2 involvement in cell invasion was less determinant than that of Kindlin-1. It was not overexpressed in breast tumors and had no prognostic value at the clinical level, suggesting that this protein is not implicated in breast cancer. Nevertheless, different studies have reported a tumor-promoting function of Kindlin-2 in this type of cancer. In particular, overexpression of Kindlin-2 in MCF7 cells was shown to drive tumor formation in mice [45]. However, since these findings were obtained with an ectopic expression of Kindlin-2, the relevance of this isoform in the clinics could not be ascertained. Other groups investigated the expression of Kindlin-2 in clinical samples [46, 47]. These studies reported an increased expression of Kindlin-2 during cancer progression (comparing benign vs malignant or in situ vs. invasive tumors). We were not able to perform such analysis in our series of breast tumors. However, Guo’s group also reported a consistent decrease of Kindlin-2 expression in cancer compared to normal breast tissue (in 45% of the tested datasets obtained from Oncomine database). These results are not in agreement with the suggested role of Kindlin-2 as a pro-tumorigenic biomarker [47].

More confusing are the phenotypic discrepancies observed between our work and cells stably inactivated for Kindlin-2 [48, 49]. Indeed, the inactivation of Kindlin-2 in MDA-MB-231 cells, using the CRISPR/Cas9 technology, induced a drastic inhibition of tumor formation, cell invasion and lung metastasis, suggesting that Kindlin-1 is not able to compensate for the loss of Kindlin-2 in these conditions.

These differences might not be relying on the transient versus stable loss of Kindlin-2. The knockout of Kindlin-2 in fibroblasts also showed de novo expression of Kindlin-1 indicating that Kindlins can effectively compensate for each other in stable cell lines [38]. One possible hypothesis that could explain the differences between both KO techniques might be relying on the limitations of CRISPR/Cas9 techniques. Researchers have recently demonstrated that CRISPR/Cas9 gene editing can cause greater genetic damage in cells than was previously thought [50]. Therefore, secondary mutations could be raised using this technique leading to a phenotype that is not completely due to the loss of the gene of interest. Unfortunately, in their reports, Sossey-Alaoui did not present the status of Kindlin-1 to verify whether or not the protein could be expressed in their Kindlin-2 breast cancer deficient cells.

Kindlin-2 has also been reported to have tumor promoting effects in several other types of cancers such as gastric and hepatocellular carcinomas [20, 51–53]. It might be due to the fact that distinct Kindlins/integrins complexes may be involved in different tissue types according to their profiles of ECM expression. To support this hypothesis, it has been shown that the repertoire of integrins expressed by cancer cells dictate the selective colonization of distinct organs [54].

However, we cannot rule out that some of the discrepancies in the role of Kindlins in different cancers might be due to the use of different experimental models and protocols as previously mentioned. In addition, in most published works on Kindlins functions mainly relying on ectopic expression or depletion of only one Kindlin isoform, the impact on the other Kindlin expression has usually not been addressed. In light of our findings emphasizing the importance of the compensatory roles of Kindlins, we suggest that these potential events should further be examined in different cancer types.

Furthermore, we found Kindlin-2 to be highly expressed by stromal breast cancer cells as previously reported in bladder and pancreatic cancers [22]. In these cancers, Kindlin-2 stromal expression was associated to cancer progression. In breast cancer, we found that Kindlin-3 expressed by stromal cells correlated with a poor patients’ outcome. Previous reports showed either an up- or a down-regulation of Kindlin-3 in breast cancer [25, 26], but we did not detect Kindlin-3 in any of our breast epithelial tumor cells. Consistent with other groups; we did not detect Kindlin-3 protein in vascular endothelial cells [55]. Kindlin-3 expressed by tumor infiltrating immune cells, was more prominent in triple-negative and ERBB2 tumors, those subtypes shown to exhibit a higher number of TILs [56, 57]. Therefore, whether Kindlin-3 expression by stromal cells might contribute to tumor progression in the same way as demonstrated for Kindlin-2 in pancreatic and bladder cancers [22] worth further investigations.

## Conclusions

Our results underline the importance of the Kindlin family in breast cancer emphasizing their redundant and specific roles in breast tumor cells. We highlight the involvement of Kindlin-1 in breast cancer progression. In addition, Kindlin-3 overexpression in the tumor microenvironment is also associated with more aggressive breast tumors.

Altogether, our work indicates that Kindlins expression may be useful in identifying breast cancer patients with a worst prognosis. Thus, our findings may offer new avenues for therapeutic intervention against cancer progression.

## Additional files

Additional file 1: **Table S1.** Clinical characteristics of the TMA population (*n* = 129). **Table S2.** PDX clinical characteristics (*n* = 58). **Table S3.** qRT-PCR primers. (PDF 170 kb)

Additional file 2: **Figure S1.** Kindlin-1 and -2 involvement in cell morphology and their subcellular localization in MDA-MB-468 and BT20 breast cancer cell lines. **Figure S2.** Kindlin-1 and Kindlin-2 involvement in breast cancer cell motility. **Figure S3.** Immune markers in the different breast cancer molecular subtypes. **Figure S4.** Kindlins mRNA levels in breast cancer molecular subtypes from METABRIC. **Figure S5.** Kindlins expression and patients’ outcome. **Figure S6.** Kindlins expressions in lung vs. bone metastasizing tumors.

## Abbreviations

ECM: Extracellular matrix
ER: Estrogen receptor
PDX: Patient derived xenograft
PR: Progesterone receptor
qRT-PCR: Quantitative reverse transcription PCR
siRNA: Small interfering RNA
TILs: Tumor infiltrating leukocytes
TMA: Tissue microarray
