# Supplementary material for: Distinct expression profiles and functions of Kindlins in breast cancer
Source: J Exp Clin Cancer Res. 2018 Nov 26;37:281. doi: 10.1186/s13046-018-0955-4 (PMC6260766; doi:10.1186/s13046-018-0955-4)
Supplement: Supplementary file 1 — Table S1. Clinical characteristics of the TMA population (n = 129). Table S2. PDX clinical characteristics (n = 58). Table S3. qRT-PCR primers. (PDF 170 kb) [file 13046_2018_955_MOESM1_ESM.pdf]

Supplementary Table 1. Clinical characteristics of the TMA population (n=129)

**Age at diagnosis**	
	55.3 years (22-83 years)
**SBR histological grade**	
I	11
II	27
III	77
**Lymph node status**	
0	85
1-3	17
>3	13
**Macroscopic tumor size**	
≤25 mm	95
>25 mm	20
**ER status**	
Negative	74
Positive	41
**PR status**	
Negative	77
Positive	38
**HER2 status**	
Negative	79
Positive	36
**Molecular subtypes**	
TNBC	61
ERBB2	16
Luminal A	17
Luminal B	21

*Information only available for 115 samples

Supplementary Table 2. PDX clinical characteristics (n=58)

**Origin**	
Primary tumor	51
Lymph node metastasis	4
Omentum metastasis	2
**Molecular characteristics**	
TNBC	38
ER	13
ERBB2	21

*Information only available for 57 PDX

Supplementary Table 3. qRT-PCR primers

**Kindlins Human-specific primers**	
Kindlin-1 (Hs-for)	5′-TGATCTTTGCAG CTCTAC AGTACCA-3′
Kindlin-1 (Hs-rev)	5′-CTTCTATTTCATCAACCTCGGACTC-3′
Kindlin-2 (Hs-for)	5’-CTGATTCCAGTTGCAGAAGGCAT-3’
Kindlin-2 (Hs-rev)	5’-CCAGTGTGCATACTGTTTTTCATTGT-3’
Kindlin-3 (Hs-for)	5’-AAGGCCGGGGACGCACTCT-3’
Kindlin-3 (Hs-rev)	5’-TCAGCCGCACGGGGTCTGT-3’
**Kindlins Mouse-specific primers**	
Kindlin-1 (Mm-for)	5’-CAAGCTCGTGGAACAGATGAACATAG-3’
Kindlin-1 (Mm-rev)	5’-CAGTGTGTTTTCAGAAGCCAGCAG-3’
Kindlin-2 (Mm-for)	5’-AAGACACGTCCATCTCCTGCTACA-3’
Kindlin-2 (Mm-rev)	5’-TCCGGAGTAACTTCACATCCTCTG-3’
Kindlin-3 (Mm-for)	5’-TCTGCCGCCTCCTCAGTATCA-3’
Kindlin-3 (Mm-rev)	5’-TTGTCAGGTCATGCACCTCCTCT-3’
**Other primers used**	
TBP (Hs-for)	5’-TGCACAGGAGCCAAGAGTGAA-3’
TBP (Hs-rev)	5’-CACATCACAGCTCCCCACCA-3’
CD45 (Hs-for)	5’-GTATTTGTGGCTTAAACTCTTGGCAT-3’
CD45 (Hs-rev)	5’-TCCAGTGGGGGAAGGTGTTG-3’
CD86 (Hs-for)	5’-TCTGAACTGTCAGTGCTTGCTAACTT-3’
CD86 (Hs-rev)	5’-AGGTTCTGGGTAACCGTGTATAGATG-3’
CD28 (Hs-for)	5’-GTGGCCTTTATTATTTTCTGGGTGAG-3’
CD28 (Hs-rev)	5’-GGCGGGGAGTCATGTTCATGT-3’
CD4 (Hs-for)	5’-AGGCGGTGTGGGTGCTGAA-3’
CD4 (Hs-rev)	5’-GACCATGTGGGCAGAACCTTGA-3’
