# Supplementary material for: Distinct expression profiles and functions of Kindlins in breast cancer
Source: J Exp Clin Cancer Res. 2018 Nov 26;37:281. doi: 10.1186/s13046-018-0955-4 (PMC6260766; doi:10.1186/s13046-018-0955-4)
Supplement: Supplementary file 2 — Figure S1. Kindlin-1 and -2 involvement in cell morphology and their subcellular localization in MDA-MB-468 and BT20 breast cancer cell lines. Figure S2. Kindlin-1 and Kindlin-2 involvement in breast cancer cell motility. Figure S3. Immune markers in the different breast cancer molecular subtypes. Figure S4. Kindlins mRNA levels in breast cancer molecular subtypes from METABRIC. Figure S5. Kindlins expression and patients’ outcome. Figure S6. Kindlins expressions in lung vs. bone metastasizing tumors. [file 13046_2018_955_MOESM2_ESM.pdf]

Supplementary Figure 1

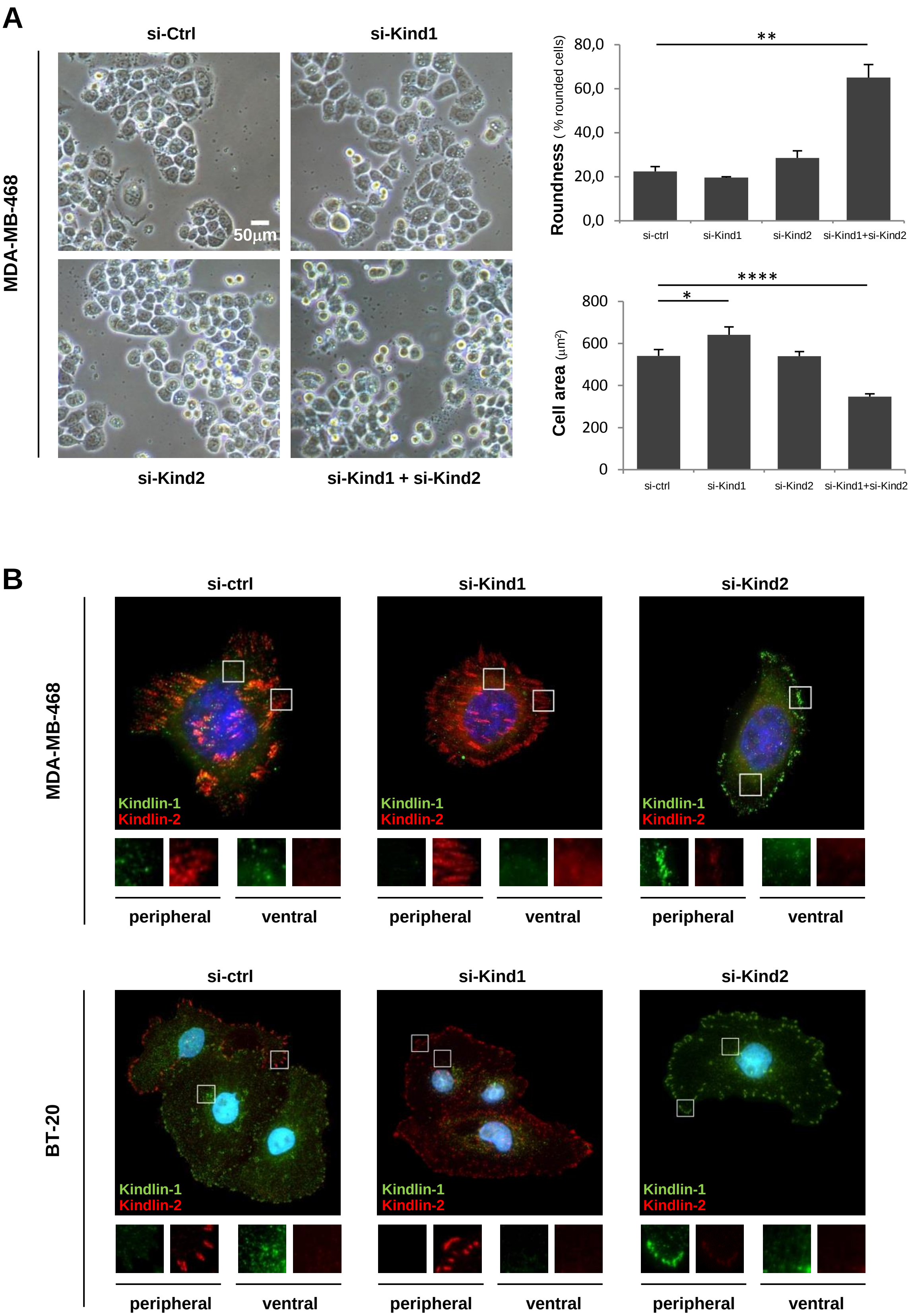

Supplementary Figure 1. **Kindlin-1 and -2 involvement in cell morphology and their subcellular localization in MDA-MB-468 and BT20 breast cancer cell lines.** BT20 and MDA MB 468 cells were transfected with control siRNA (si-ctrl), *KIND1* siRNA (si-Kind1), *KIND2* siRNA (si-Kind2) alone or in combination (si-Kind1+si-Kind2). **(A)** Phase contrast microscopy was performed in MDA-MB-468 cells to calculate the roundness (% of rounded cells) and cell area (μm^2) represented as the mean +/- SEM of values. Statistical analysis were performed by a *t-test* (*$****p<0.0001$; $**p<0.01$; $*p<0.05$*). **(B)** Five days after transfection, MDA-MB-468 and BT20 cells were fixed, permeabilized and immunostained with anti-Kindlin-1 and anti-Kindlin-2 antibodies. Cells were then counterstained with DAPI and imaged with a fluorescence microscope (original magnification: X100).

Supplementary Figure 2

A

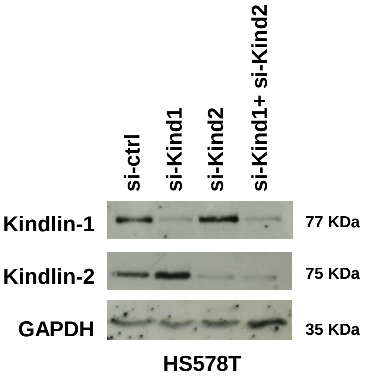

B

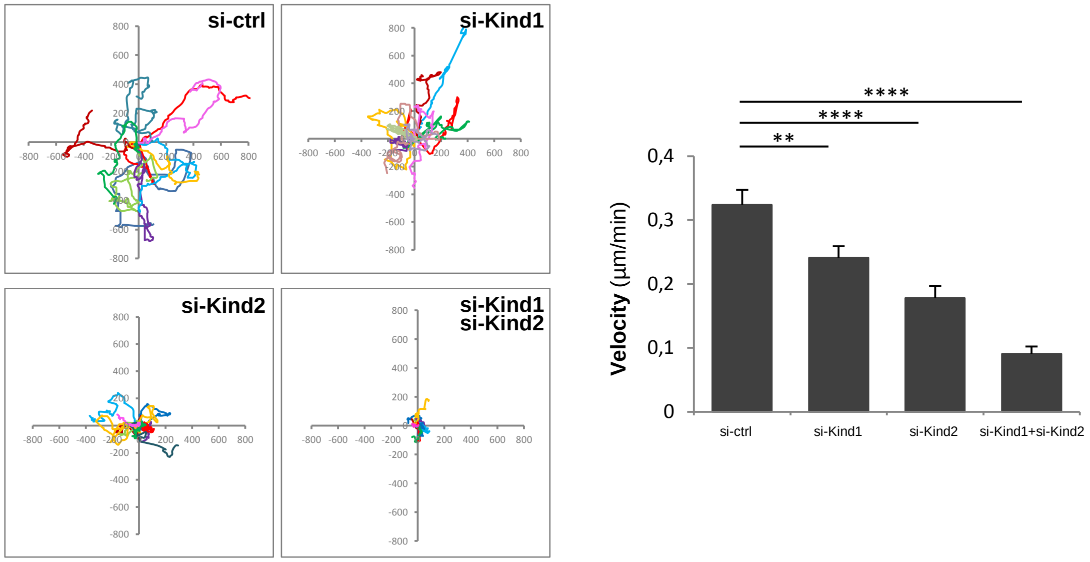

C

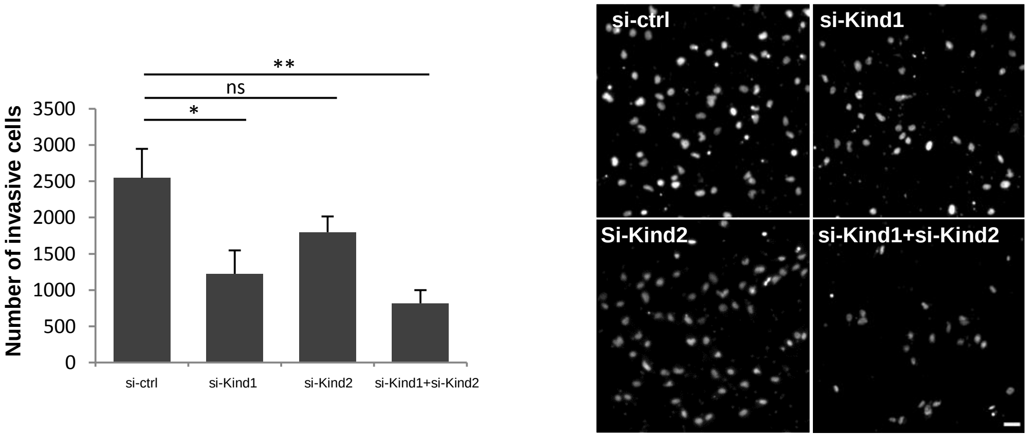

Supplementary Figure 2. Kindlin-1 and Kindlin-2 involvement in breast cancer cell motility. Hs.578T cells were transfected with control siRNA (si-ctrl), *KIND1* siRNA (si-Kind1), *KIND2* siRNA (si-Kind2) alone or in combination (si-Kind1+si-Kind2) for five days. **(A)** Cellular extracts were immunoblotted with anti-Kindlin-1, anti-Kindlin-2, and anti-GAPDH (loading control) antibodies. **(B)** Time-lapse imaging was performed for 24h. Plots show overlays of the representative trajectories travelled by cells. Velocity was quantified and represented as the mean +/- SEM of values (n=30 cells tracked by condition). Statistical analysis were performed by a *t-test* (*****$p<0.0001$; **$p<0.01$*). Results are representative of experiments performed at least in duplicate. **(C)** A transwell cell invasion assay was performed for 24h. Cells were then counterstained with DAPI and imaged with a fluorescence microscope. The number of invasive cells was quantified and represented as the mean ± SEM of values. Statistical analyses were performed by a *t-test* (***$p<0.01$; *$p<0.05$;* ns: not significant). A representative image of three independent experiments is shown for each condition. Scale bar = 50 µm.

Supplementary Figure 3

A

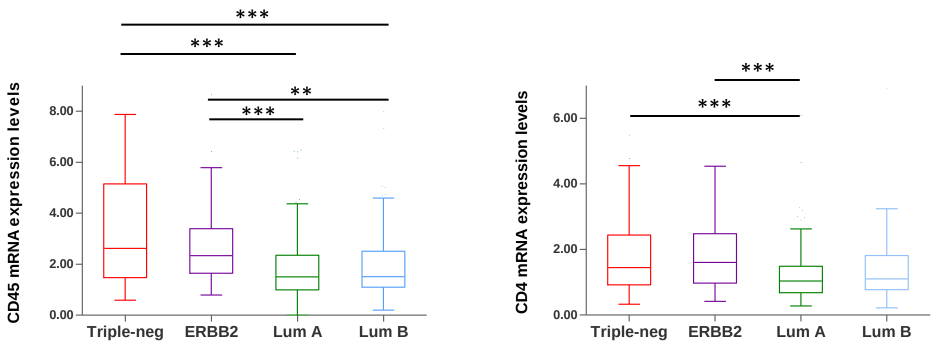

B

Spearman's correlation test

		Kindlin-3	
**Immune marker**		r	p-value
CD45	Hematopoietic cells	**0,699**	<0,0000001
CD86	Antigen presenting cells	**0,677**	<0,0000001
CD28	T-cells	**0,639**	<0,0000001
CD4	Helper T-cells	**0,797**	<0,0000001

Supplementary Figure 3. Immune markers in the different breast cancer molecular subtypes. **(A)** Box-and-Whisker plot showing CD45 and CD4 mRNA expression levels in a series of 438 breast cancer patients grouped according to four well described breast tumor molecular subtypes (Triple negative, ERBB2, Luminal A and Luminal B). Statistical analysis were performed by a Tukey's multiple comparison *test (***p<0.001; **p<0.01*). **(B)** Spearman's correlation test measuring the strength and significance of the association between Kindlin-3 and different immune marker expressions (r: correlation coefficient).

Supplementary Figure 4

A

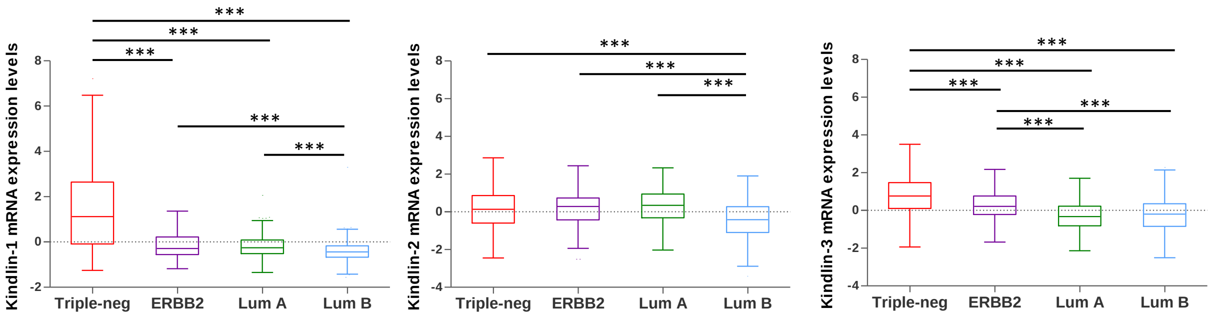

B

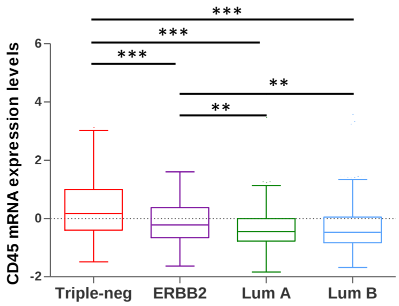

C

Spearman's correlation test

		Kindlin-3	
**Immune marker**		r	p-value
CD45	Hematopoietic cells	**0,592**	<0,0001
CD86	Antigen presenting cells	**0,834**	<0,0001
CD28	T-cells	**0,517**	<0,0001
CD4	Helper T-cells	**0,866**	<0,0001

Supplementary Figure 4. Kindlins mRNA levels in breast cancer molecular subtypes from METABRIC. **(A)** Box-and-Whisker plot showing kindlin-1, -2 and -3 mRNA expression levels in a series of 2509 breast cancer patients grouped according to four well described breast tumor molecular subtypes (Triple negative, ERBB2, Luminal A and Luminal B). **(B)** Box-and-Whisker plot showing CD45 mRNA expression levels in the same series of breast tumor. Statistical analysis were performed by a Tukey's multiple comparison *test (***p<0.001; **p<0.01)*. **(C)** Spearman's correlation test measuring the strength and significance of the association between Kindlin-3 and different immune marker expressions (r: correlation coefficient).

Supplementary Figure 5

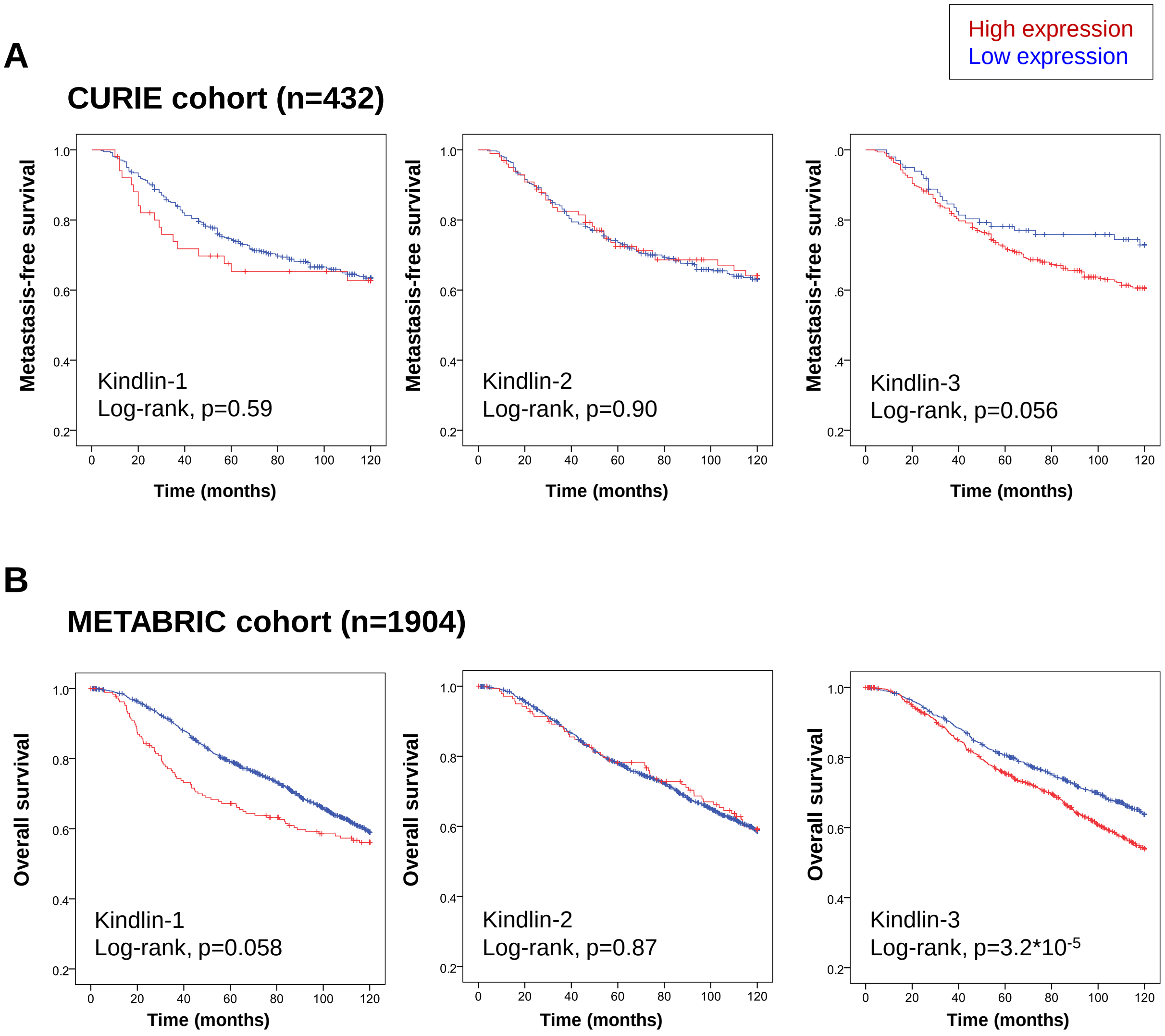

Supplementary Figure 5. Kindlins expression and patients' outcome. (A) Kaplan-Meier curves showing overall metastasis-free survival (within the first 120 months after breast cancer diagnosis) of patients with tumors expressing high (red lines) vs. low (blue lines) levels of Kindlins analyzed by qRT-PCR. **(B)** Kaplan-Meier curves showing overall survival (within the first 120 months after breast cancer diagnosis) of patients from METABRIC with tumors expressing high (red lines) vs. low (blue lines) levels of Kindlins mRNAs. Statistical analyses were performed by a Log-rank *test*.

Supplementary Figure 6

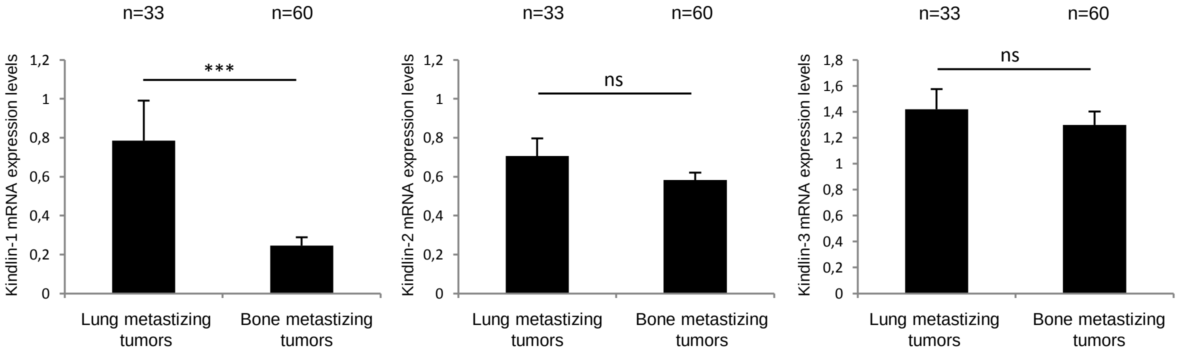

Supplementary Figure 6. Kindlins expressions in lung vs. bone metastasizing tumors. Comparison of Kindlin-1, -2 and -3 mRNA expression levels in tumors metastasizing exclusively to lungs (n=33) vs. bones (n=60) within the first 150 months after breast cancer diagnosis. mRNA levels were determined by qRT-PCR (Mean+SEM represented, ****p<0.001*, ns: not significant, *t-test*).
